# Influence of the Core Formulation on Features and Drug Delivery Ability of Carbamate-Based Nanogels

**DOI:** 10.3390/ijms21186621

**Published:** 2020-09-10

**Authors:** Filippo Pinelli, Fabio Pizzetti, Óscar Fullana Ortolà, Alessandro Marchetti, Arianna Rossetti, Alessandro Sacchetti, Filippo Rossi

**Affiliations:** Department of Chemistry, Materials and Chemical Engineering “Giulio Natta”, Politecnico di Milano, via Mancinelli 7, 20131 Milan, Italy; filippo.pinelli@polimi.it (F.P.); fabio.pizzetti@polimi.it (F.P.); oscar.fullana@mail.polimi.it (Ó.F.O.); alessandro.marchetti@polimi.it (A.M.); arianna.rossetti@polimi.it (A.R.)

**Keywords:** nanogel, polymers, drug release, tunability

## Abstract

In the last years, nanogels have emerged as one of the most promising classes of novel drug delivery vehicles since they can be employed in multiple fields, such as various therapeutics or diagnostics, and with different classes of compounds and active molecules. Their features, such as a high volume to surface ratio, excellent drug loading and release ability, as well as biocompatibility and tunable behavior, are unique, and, nowadays, great efforts are made to develop new formulations that can be employed in a wider range of applications. Polyethylene glycol (PEG)-polyethylenimine (PEI) nanogels probably represent the baseline of this class of biomaterials and they are still largely employed and studied. In any way, the possibility to exploit new core formulations for nanogels is certainly very interesting in order to understand the influence of different polymer chains on the final properties of the system. In this research, we explore and make a comparison between PEG-PEI nanogels and two other different formulations: pluronic F127-PEI nanogels and PEG-Jeffamine nanogels. We propose nanogels synthesis methods, their chemical and physical characterization, as well as their stability analysis, and we focus on the different drug delivery ability that these structures exhibit working with different typologies of drug mimetics.

## 1. Introduction

In the panorama of drug delivery systems, nanogels surely represent one of the most important families of devices [1,2,3]. They are commonly identified as a nano-system made of an aqueous dispersion of hydrogel particles obtained through a chemically or physically crosslinking network [4,5]. The main features of nanogels, such as nanometric dimensions, swelling behavior, high stability, great drug loading, and release ability, made them an ideal candidate for drug delivery applications [6,7,8]. Many references are available in literature that testify the dissemination that nanogels have acquired in the nanomedicine field and in the treatment of various pathologies, confirming the efficacy they have in respect to conventional treatments (e.g., improved pharmacokinetics, precise targeting, reduced side effects) [4,9] and their potential in stimuli-responsive targeted therapies (e.g., thermoresponsive nanogels) [10,11].

This kind of device was introduced for the first time by Vinogradov et al. in 1999, describing a hydrophilic polymeric network obtained through crosslinking between polyethylene glycol (PEG) and polyethylenimine (PEI) [12]. In the past years, many studies have been realized on the formulations of these structures and two important approaches have been adopted [13,14,15]. On one side, it is possible to change the formulation of the core structure of nanogels, working with different polymers, tuning their quantities, and adopting a proper synthesis method to enhance the crosslinking degree [13,16,17,18,19,20]. Sabatino et al. [13] demonstrated that with a proper selection of the irradiation conditions, polymer concentration, and gaseous atmosphere, nanogels with the desired features in terms of dimensions, surface electric charge, and chemical reactivity can be produced. In particular, radiation-engineered poly (*N*-vinyl pyrrolidone)-based nanogels bearing carboxyl groups and primary amines can be used as the main building block of promising nanodevices. Li et al. [19] studied the possibility to construct polyion complex micelles made of copolymers and cholic acid and to evaluate the potential of this type of micelles as a targeted drug delivery system for paclitaxel. To further improve the targeting capability of micelles, folate was also incorporated into micelles (core part). In a similar direction, Moya-Ortega and coworkers [20] reviewed many possibilities to incorporate hydrophilic cyclodextrin moieties into the polymeric network of the nanogels. This strategy is able to provide them with a drug loading and release mechanism that is based on the formation of inclusion complexes without decreasing the hydrophilicity of the network. Similarly, Argenziano et al. [21] developed polyamidoamines nanogels with the insertion of cyclodextrin moieties that add hydrophobic drug-complexing sites. These devices were designed for the delivery of dexamethasone, and in vitro studies demonstrated the efficacy of this formulation, showing faster action with lower doses and reduced side effects.

On the other side, it is possible, maintaining the same core structure of the system, to functionalize the surface of the nanogels with various coating strategies [22,23].

In this direction, Papa and coworkers introduced an amine group to PEG-PEI nanogels to increase astrocytes selectivity for spinal cord injury treatment [24]. This strategy can be also extremely useful to reduce macrophage uptake that is undesired in many medical problems [25]. Details about this strategy can be found in a recent review published by our group [22].

In each case, the formulations can tune the properties or even the biological behavior of the system, making it proper to specific drug delivery applications [26,27].

In the light of this, in this research, we have decided to focus on the core formulations of nanogels and we have realized three different typologies of nanogels. First of all, we synthetized PEG-PEI nanogels (PEG-PEI NGs), which represent the baseline of this family of biomaterials. Then, we investigated what happens if one of the two constituent polymers is substituted with a polymeric chain with different properties [28,29].

Afterwards, we fabricated pluronic F-127-PEI nanogels (Plu-PEI NGs), working with a similar synthesis method, but exploiting pluronic polymer instead of the PEG chain [19,30]. We propose here the activation strategy for pluronic F-127, analyzing the functionalization of the final product [31].

Alongside, we also realized the PEG-Jeffamine nanogels (PEG-Jef NGs). Similarly to what we have done with Plu-PEI NGs, we substitute here the PEI chains of PEG-PEI NGs with Jeffamine (Elastamine RE-2000) to obtain the final nano-system.

These three different formulations of nanogels have been characterized both chemically, through ^1^H-NMR spectra, and physically, through DLS analysis on their size, stability, and surface potential, to understand the influence of the employed polymeric chains on the properties of the final system; in addition, their biocompatibility was investigated as well. Moreover, we focused on drug delivery ability of these devices, investigating their features in terms of drug loading and drug release, working with three different drug mimetics both hydrophilic and hydrophobic [32,33,34,35].

## 2. Materials and Methods

### 2.1. Materials

The experiments were performed using the following polymers: polyethylene glycol 8000 (MW = 8000 Da, by Sigma-Aldrich Chemie GmbH, Deisenhofen, Germany), polyethyleneimine linear (MW = 2500 Da, by Polysciences Inc., Warrington, PA, USA), pluronic^®^ F-127 powder (MW = 12,600 Da, by Sigma-Aldrich Chemie GmbH, Deisenhofen, Germany), and elastamine^®^ RE-2000 amine (MW = 2000 Da, Huntsman Corporation, Ternate, Italy) as polyetherdiamine. All other used chemicals were purchased from Sigma-Aldrich (Sigma-Aldrich Chemie GmbH, Deisenhofen, Germany). The materials were used as received; solvents were of analytical grade. Products containing rhodamine, fluorescein sodium salt, or oil red O were stored at 4 °C in the dark.

### 2.2. Synthesis of PEG-PEI Nanogels

The synthesis of PEG-PEI nanogels has been widely discussed in literature and in our previous studies (please see the Supplementary Materials file) [36,37,38]. Briefly, the first two preliminary steps of the procedure are the functionalization of PEG polymer and PEI polymer. The activation of PEG 8000 hydroxyl groups is realized working with 1,1′-carbonyldiimidazole (CDI). Then, nanogel formation is realized through the modified emulsification evaporation method, as discussed by Vinogradov and coworkers [12]. The activated PEG (200 mg, 0.025 mmol) was dissolved in dichloromethane (3 mL), while PEI (50 mg, 0.02 mmol) was dissolved in distilled water (3 mL). Then, the organic solution was added dropwise, under vigorous stirring, to PEI solution, and the resulting mixture was sonicated for 30 min. The system was allowed to stir for 17 h at room temperature (25 °C) promoting the evaporation of CH_2_Cl_2_.

### 2.3. Synthesis of Pluronic F-127-PEI Nanogels

Pluronic F-127-PEI nanogels were synthetized following the same procedure illustrated in the previous section, using activated pluronic F-127 instead of activated PEG.

Briefly, pluronic F-127 was activated through 1,1′-carbonyldiimidazole (CDI) with the same strategy used with PEG, as discussed in literature [19,39,40]. Then, the activated pluronic F-127 (200 mg, 0.015 mmol) was dissolved in dichloromethane (3 mL) and this organic solution was added dropwise, under vigorous stirring, to a solution of PEI (50 mg, 0.02 mmol) in distilled water (3 mL). Then, the resulting mixture was sonicated for 30 min and the system was allowed to stir for 17 h at room temperature (25 °C) promoting the evaporation of CH_2_Cl_2_.

### 2.4. Synthesis of PEG-Jeffamine Nanogels

Similarly to the synthesis of Plu-PEI NGs, PEG-Jeffammine nanogels were realized following the same procedure illustrated for PEG-PEI nanogels, using Elastamine RE-2000 instead of activated PEI [41]. The activated PEG (96 mg, 0.012 mmol) was dissolved in dichloromethane (3 mL), while Elastamine RE-2000 (240 mg, 0.12 mmol) was dissolved in 3 mL of water. The resulting mixture was sonicated for 30 min and the system was allowed to stir for 17 h at room temperature (25 °C) promoting the evaporation of CH_2_Cl_2_.

### 2.5. Chemical Characterization of Polymers and Nanogels

The NMR analyses were carried out on a Brucker AC (400 MHz). Polymers functionalization, CDI functionalization on PEG and pluronic F-127 polymers, were verified through comparisons with literature references. Nanogel frameworks were chemically characterized through NMR analyses using D_2_O as solvent.

### 2.6. Physical Characterization of Nanogels

The hydrodynamic diameters of the synthetized nanogels and their ζ-potential were estimated through dynamic light scattering (DLS) measurements using a Zetasizer Nano ZS from Malvern Instruments. The reported data are the result of an average value of three measurements of the same sample. Size measurements were realized in distilled water, while ζ-potential estimations were realized in phosphate-buffered saline.

Moreover, we analyzed the variations of the size values of the nanogels to investigate the degradation of the framework of the system. In order to perform this evaluation, we analyzed the same sample every two days for at least ten days, observing the variations in the measured hydrodynamic diameters. During these tests, the samples were stored at 37 °C to simulate biological conditions.

### 2.7. Loading of Nanogels with Different Drug Mimetics

Three different drug mimetics were employed in drug loading and delivery tests. Sodium fluorescein (SF) and rhodamine B (RhB) were used as hydrophilic drug mimetics, while pyrene was used as hydrophobic drug mimetic [41,42,43].

Regarding SF and RhB, the same procedure was performed for the loading of nanogels: a drug mimetic solution was prepared dissolving, separately, SF and RhB in distilled water (1 mg/mL). Lyophilized nanogels (PEG-PEI NGs, Plu-PEI NGs, PEG-Jef NGs) were independently suspended in aqueous solution (20 mg/mL). Then, 1 mL of drug mimetic solution was added dropwise (1 mL/min) to 1 mL of nanogel solution under stirring and the system was left to stir for 17 h at 25 °C in the dark. In this way, the loading of SF and RhB for each type of synthetized nanogel was performed.

In the case of the loading of pyrene in nanogels, a drug mimetic solution dissolving pyrene in dimethyl sulfoxide (5 mg/mL) was prepared. Lyophilized nanogels (PEG-PEI NGs, Plu-PEI NGs, PEG-Jef NGs) were independently suspended in dimethyl sulfoxide (20 mg/mL). Then, 50 μL of drug mimetic solution was added dropwise to 1 mL of nanogel solution under stirring, and the system was left to stir for 17 h at 25 °C in the dark. This process with pyrene was realized for each type of synthetized nanogel.

### 2.8. Drug Loading Evaluation and In Vitro Drug Delivery

The drug release mechanism was investigated at pH 7.4 using a phosphate-buffered saline solution (PBS). In detail, each nanogel sample was placed in excess of PBS, and aliquots (3 × 100 μL) were collected at defined time points, while the sample volume was replaced by a fresh solution, in order to avoid mass-transfer equilibrium with the surrounding release environment. The experiments were performed at 37 °C. Percentages of released hydrophilic drug molecules (rhodamine B, sodium fluorescein) and a hydrophobic one (pyrene) were then measured by UV spectroscopy at a specific wavelength. In the cases of SF (*λ* = 485 nm) and RhB (*λ* = 570 nm), aliquots were directly analyzed; for the test with pyrene (*λ* = 334 nm), the aliquots were lyophilized and then solved in acetonitrile before being analyzed [42]. Loading efficiency (% loading) was calculated referring to Equation (1):

(1) $$\%\;loading=\frac{drug\;entrapped\;within\;NGs}{initial\;amount\;loaded}\cdot 100.$$

The drug diffusion mechanism can be described as a 1-dimensional model of the second Fick law [37] where the nanogel geometry is a sphere and the material flux mainly takes place at the PBS/nanogel surface. Equation (2) shows these considerations, indicating *r* as the characteristic radius for the mass transport phenomenon. The following mass balance equations (Equation (3)) are written considering the variation of the mean drug concentration within the nanogel (*C_G_*) related to the volume of solution (*V_S_*), the mean drug concentration in the outer solution (*C_S_*), the total volume (*V_G_*), the NGs mass present inside the matrix (m_G_), and the exchange interfacial surface (*S_exc_*) that represents the boundary surface between the nanogel and the surrounding solution (which, simplifying, can be here considered as being only the side surface). Equations (4) and (5) describe the mean drug concentration in the outer solution (*C_S_*) and the mean drug concentration within the nanogel (*C_G_*) at the initial conditions (*t* = 0). According to these expressions, the boundary conditions are defined describing the profile symmetry at the center of the polymeric sphere, with respect to the radial axis of the sphere (Equation (6)) and the equivalence between the material diffusive fluxes at the PBS/nanogel surface (Equation (7)).

(2) $$\frac{\partial C_{G}}{\partial t}=D\times \frac{1}{r^{2}}\times \frac{\partial }{\partial r}\times \left(r^{2}\times \frac{\partial C_{G}}{\partial r}\right)$$

(3) $$V_{S}\times \frac{\partial C_{G}}{\partial t}=k_{C}\times S_{exc}\times \left(C_{G}-C_{S}\right)$$

(4) $$C_{S}\left(t=0\right)=0$$

(5) $$C_{G}\left(t=0\right)=C_{G,0}=\frac{m_{G,0}}{V_{G}}$$

(6) $${\frac{\partial C_{G}}{\partial t}|}_{r=0}=0$$

(7) $$-D\times {\frac{\partial C_{G}}{\partial r}|}_{r=R}=k_{C}\times \left(C_{G}-C_{S}\right)$$

This mathematical model allowed to estimate the diffusion coefficient (*D*) of drug mimetics in order to evaluate the influence of the nanogel on the drug release. The Sherwood number obtained by means of the penetration theory (Equation (8)) allowed the computation of the mass transfer coefficient *k_C_*:

(8) $$Sh=2=\frac{k_{C}\times 2r}{D}$$

### 2.9. Primary Cell Cultures

Primary culture of microglia cells was obtained from the spinal cord of 13-day-old C57 BL/6J mouse embryos as previously described in literature [37]. Briefly, spinal cords were dissected, exposed to DNAse and trypsin, and centrifuged through a BSA cushion. Cells obtained were a population of mixed neuron/glia. These underwent centrifugation through a 6% iodixanol (OptiPrepTM) cushion to separate large neurons from glial cells. The glial fraction was cultured at a density of 25,000 cells/cm^2^ into 75 cm^2^ flasks, previously pre-coated with poly-l-lysine. Isolated microglia were obtained from flasks containing confluent mixed glial cultures after overnight shaking at 275 rpm in incubators. The supernatants (containing microglia) were collected and seeded at a density of 20,000 cells/cm^2^ into 24-well plates. NGs (0.05% *w*/*v*) were then added to cell cultures for up to 3 days.

### 2.10. Biocompatibility Tests

In order to verify the biocompatibility of the synthetized devices, the different nanogels (0.05% *w*/*v*) were added to microglia cell cultures for up to 3 days. After 3 days, the cytotoxicity of the different nanogels was evaluated by running a MTS assays and LDH release, measuring the absorbance at 570 nm for the case of the MTS assay and at 490 nm for the case of LDH release. In order to determine the relative cell viability, the results were compared with those of the control wells.

## 3. Results and Debate

### 3.1. Nanogel Chemical Characterization

**Figure 1:**
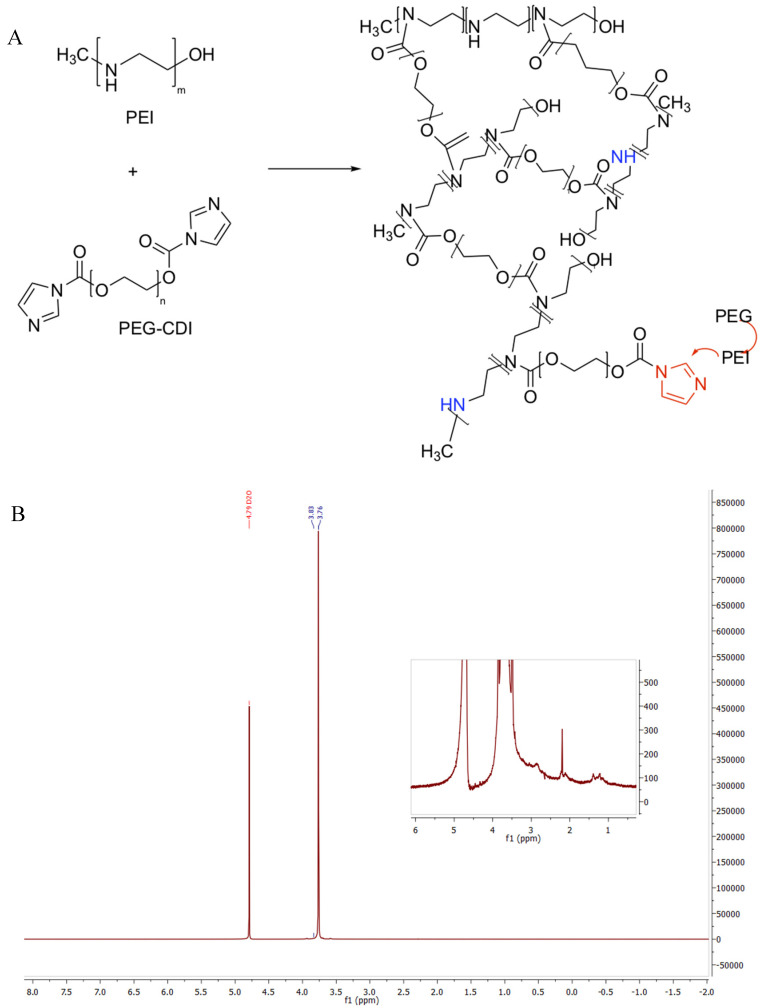
Reaction scheme of polyethylene glycol (PEG)-polyethylenimine (PEI) nanogels (NGs) formation (**A**) and its ^1^H-NMR spectrum (**B**).

**Figure 2:**
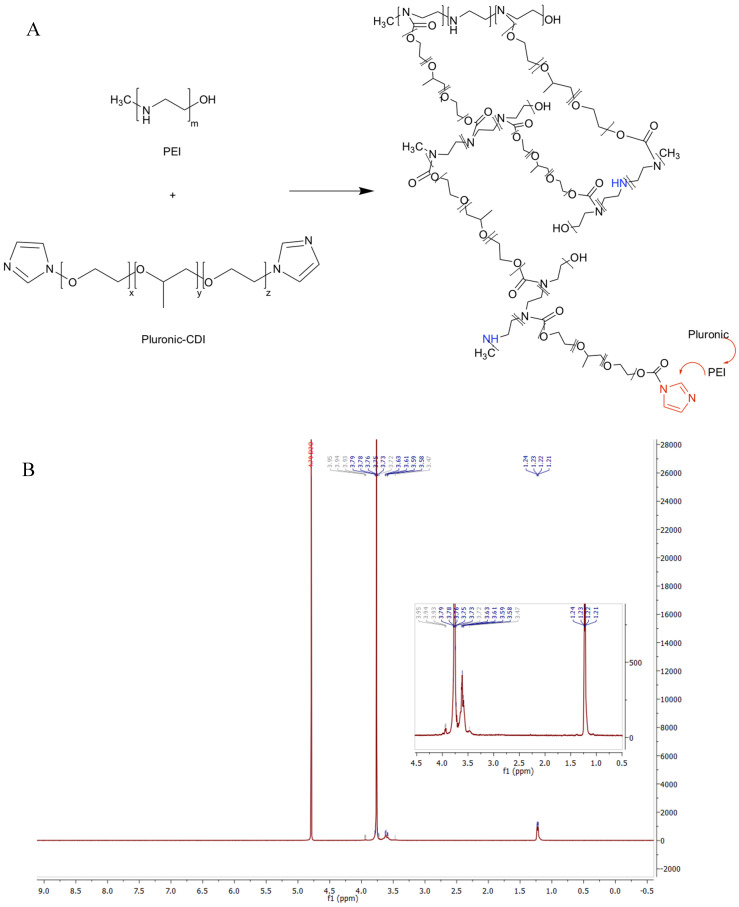
Reaction scheme of pluronic (Plu)-PEI NGs formation (**A**) and its ^1^H-NMR spectrum (**B**).

**Figure 3:**
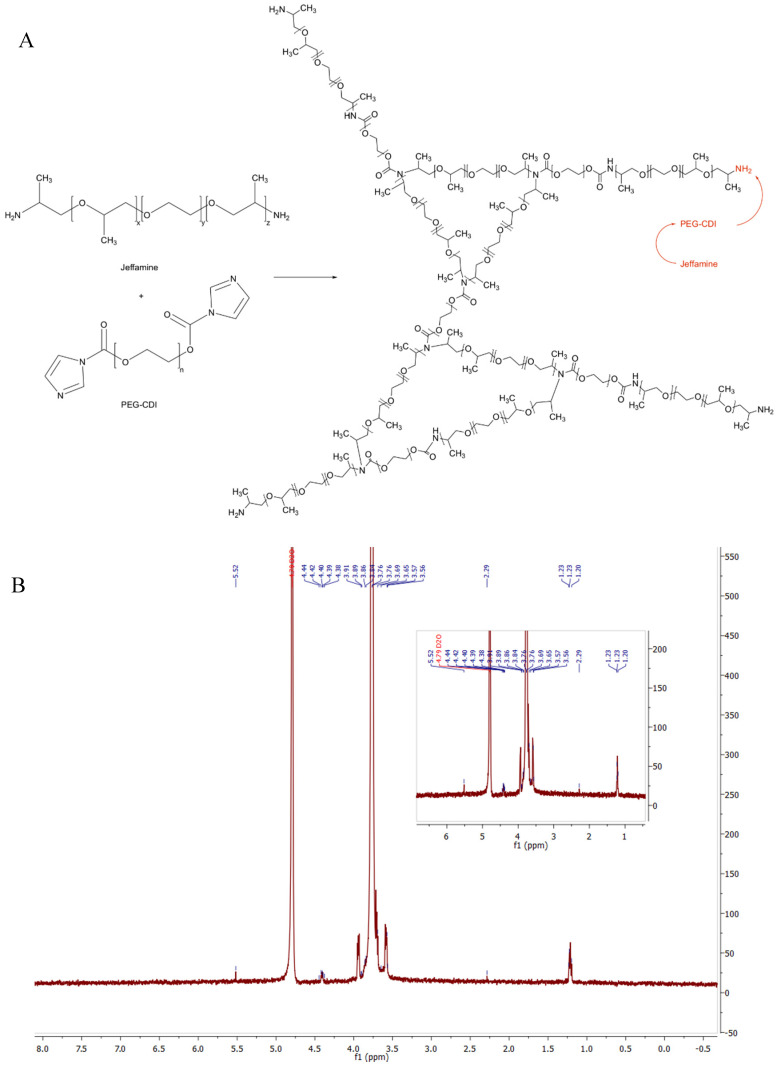
Reaction scheme of PEG-Jeffamine (Jef) NGs formation (**A**) and its ^1^H-NMR spectrum (**B**).

**Figure 4:**
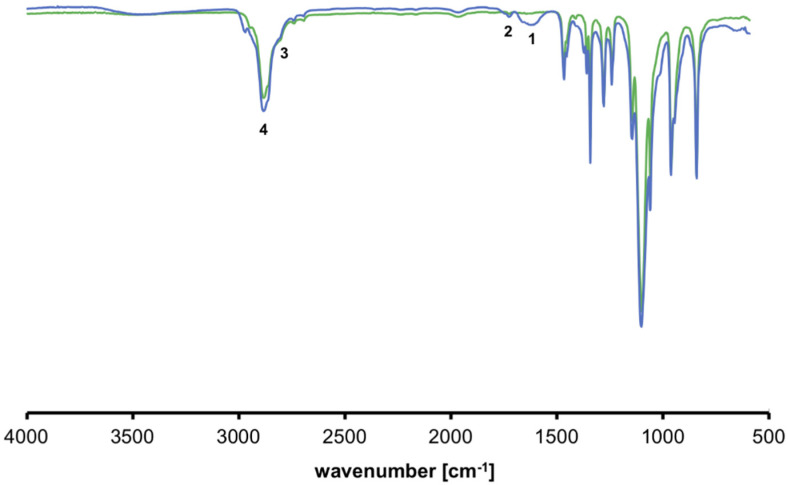
FTIR spectra of Plu-PEI (blue) and PEG-Jef (green).

The synthesis of nanogels was performed with the procedures described in the previous section working with proper functionalized polymers. As aforementioned, the functionalization of the polymeric chains was verified through NMR analysis and confirmed by the comparisons of their spectra with the results of previous literature research. Similarly, we chemically characterized the final nanogel framework through the ^1^H-NMR spectrum and we verified their formation as represented in Figure 1 for PEG-PEI NGs, in Figure 2 for Plu-PEI NGs, and in Figure 3 for PEG-Jef NGs. In Figure 4, FT-IR characterization of Plu-PEI and PEG-Jef nanogels is reported and compared with PEG-PEI NGs already published [18]. All three NGs present similar peaks due to the common nature of the components. In particular, they show the characteristic signals of PEG, PEI, pluronic, and Jeffamine chains: the wavenumbers range around 1470–800 cm^−1^ shows peaks related to PEG and Pluronic chains, as well as C-N stretching and C-H bending of PEI and Jeffamine, while a peak around 1600 cm^−1^ (1) is due to N-H bending. At 1715 cm^−1^ (2), a characteristic stretching vibration of C-O is represented (carbamate group responsible of NGs formation). The signals at 2730 cm^−1^ (3) and at 2900 cm^−1^ (4) are respectively due to the N-H and C-H stretch of the polymers.

### 3.2. Nanogel Physical Characterization

The nanogel were characterized through dynamic light scattering analyses. The results are illustrated in Table 1 comparing the three different nanogels structures in term of size and ζ-potential.

The analyses confirm that all the samples have nanometric dimensions that enable potential cellular internalization with variations in the hydrodynamic diameter related to the interaction between nanoparticles and the external medium. Indeed, pluronic, which is the most hydrophobic polymer used, causes a drastic reduction in the NGs diameter. Therefore, it is confirmed that by maintaining PEI chains, while changing the hydrophilicity of the other polymer, we can tune NGs dimensions. In particular, the higher the hydrophobicity, the smaller the diameter. The same results were confirmed with AFM analysis. Moreover, each structure presents a ζ-potential value around the neutrality, confirming that in each case, the formulation of the nanogel determines on the final system a neutral surface charge distribution. In addition, we can also observe that the polydispersity index (PDI) value depends on the polymer used, and being NGs, the trend is the opposite: the higher the hydrophilicity, the lower the PDI due to the good interactions between polymer and solvent.

**Figure 5:**
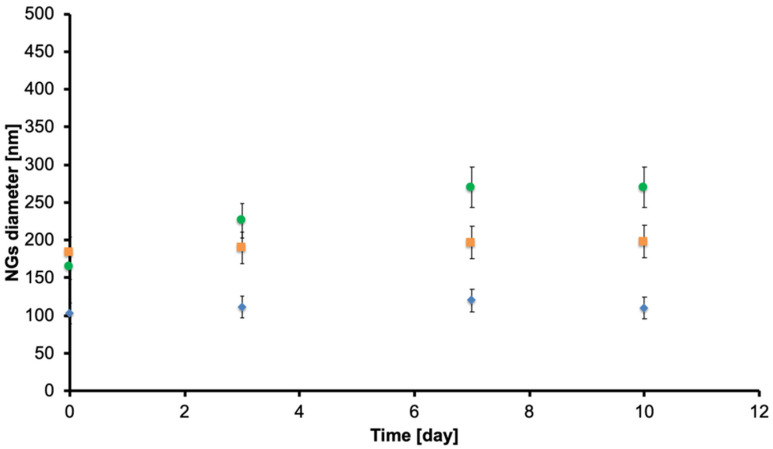
Variations in time (day) of hydrodynamic diameter (nm) of PEG-PEI NGs (orange), Plu-PEI NGs (blue), and PEG-Jef NGs (green). All NGs show a stable trend in time.

In Figure 5, the variation in time of the hydrodynamic diameter of the different nanogels is reported. Each typology of NGs, PEG-PEI NGs (orange), Plu-PEI NGs (blue), and PEG-Jef NGs (green), show a stable trend in time, and because of this, their frameworks do not degrade in this time span.

### 3.3. Drug Loading and Release Profiles

In order to verify whether these nanogel systems are able to encapsulate and tune the delivery of drugs with different properties, we loaded within them three drug mimetics commonly used in literature [41,42,43]. We used sodium fluorescein (SF) and rhodamine B (RhB) as hydrophilic drug mimetic and pyrene as hydrophobic drug mimetic. We used two different hydrophilic drug mimetics to compare an ionic (SF) and a neutral (RhB) drug.

In Table 2, we report the loading percentage in nanogel structures obtained working with the three drug mimetics.

The obtained results, working with SF and RhB, showed similar values and confirm a good degree of encapsulation inside all typologies of nanogels. These results are in accordance with previous studies that involve this type of NGs [38]: the hydrophilicity is the main responsible of different drug loading. Therefore, it is consequent that increasing drug hydrophobicity loading percentages can be higher due to the extremely low affinity of the drug molecules with an aqueous environment.

Indeed, the difference between SF and RhB is in this direction: SF is more hydrophilic and so the drug loading percentage is lower. It is also possible to observe that for all the nanogels tested, the loading percentage is higher for pyrene than in the previous cases, and these values exceed 90%. In fact, pyrene is a hydrophobic drug, and thus its solubility in the aqueous washing medium is strongly limited and the percentage of loading increases.

Moreover, PEG–Jef NGs have shown the higher loading percentage in all cases, demonstrating a very good loading capacity due to the best interactions between the framework and the encapsulated molecules.

**Figure 6:**
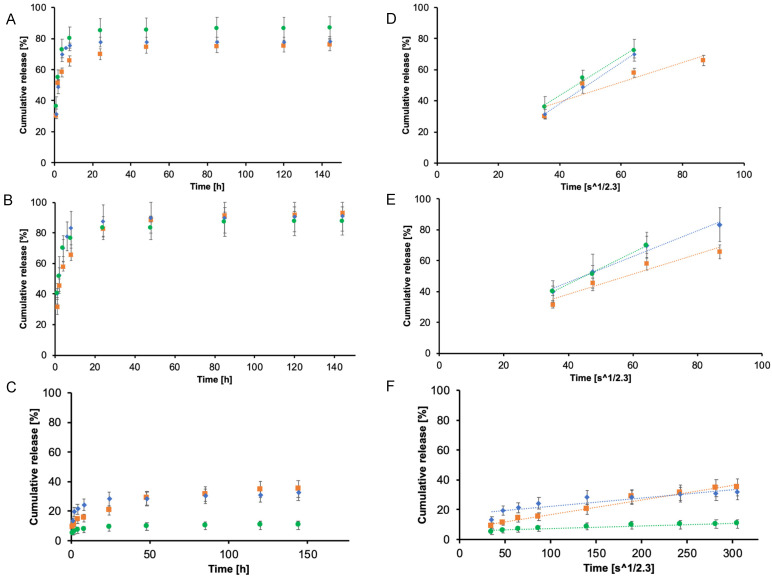
In vitro release profile of SF (**A**), RhB (**B**), and pyrene (**C**) at pH = 7.4 from PEG-PEI NGs (orange), Plu-PEI NGs (blue), and PEG-Jef NGs (green). The slope of SF release (**D**), RhB release (**E**), and pyrene release (**F**) at pH = 7.4 from PEG-PEI NGs (orange), Plu-PEI NGs (blue), and PEG-Jef NGs (green) against the variable time expressed as t^1/2.3^ is representative of the Fickian diffusion coefficient of drugs in NGs. The values are calculated as a percentage with respect to the total mass loaded (mean value ± standard deviation is plotted).

Once we have evaluated the drug loading capacities of nanogels, we started to investigate their drug release ability. The evaluation of the released amount of mimetic drug was evaluated through the UV-spectroscopy, as aforementioned in the previous section, and the percentage of released drug was defined as the ratio between the released quantities and the total amount of mimetic drug loaded in the system. In Figure 6, the release profiles of the three different nanogels loaded with each different drug mimetic are represented as the ratio between the released amount in the external medium and the amount effectively loaded inside different formulations of NGs. The release profiles of PEG-PEI NGs (orange), Plu-PEI NGs (blue), and PEG-Jef NGs (green) loaded with RhB are reported in Figure 6A; in Figure 6B, the same release profiles are reported for SF; and in Figure 6C, for pyrene. In all cases, the drug release presented a biphasic pattern with an initial burst release followed then by a sustained release. These profiles are well evident in the cases of RhB and SF, while with pyrene, the diffusion in the external aqueous medium is strongly limited by the hydrophobicity of the drug. Moreover, from the release profiles, it can be noticed that there are no remarkable differences between RhB and SF release, underlining that different NGs formulations are not heavily affecting the superficial electric charges of colloids. Instead, pyrene release exhibits a very low rate because of its hydrophobic nature. For this drug mimetic, PEG-PEI NGs and Plu-PEI NGs show very similar release profiles, while the release from PEG-Jef NGs is strongly limited, probably due to the best encapsulation and interactions with pyrene that is guaranteed by this NGs framework.

The influence of the systems in delivering drug mimetics was investigated plotting release percentage against the time to the power of 0.43 (t^1/2.3^ in Figure 6D–F). A linear plot is representative of Fickian diffusion and the y-axis intercept value is an indication of burst release, where it is well-known that an ideal controlled release system should present a linear trend in time and its y-axis intercept should be equal to zero.

The releases of RhB and SF from NGs showed a linear trend for the first six to eight hours for each nanogel formulation, while the release of pyrene showed a linear trend for all nanogels for the whole time interval. These trends can be explained considering the hydrophobicity of pyrene and the subsequent very slow release in time where the two main contributions are the diffusional one and the low solubility of the drug in aqueous media. Moreover, even in this case, considering the release of pyrene, PEG-PEI NGs and Plu-PEI NGs show a similar behavior with comparable linear profiles, while PEG-Jef NGs exhibits a reduced release, with a less steep profile probably due to the best interactions of the nanogel framework with this drug mimetic that, as we have explained before, limits its release.

Finally, we estimated the diffusion coefficient (D) of the drug mimetics in order to evaluate the influence of the framework on the drug release. The calculation was possible only with SF and RhB because of the low amount of pyrene released that is driven not only by diffusion but also by the low solubility of the drug in the aqueous media. This evaluation was performed on RhB and SF and the obtained results are reported in Table 3. Results are in accordance with drug delivery studies [34].

### 3.4. Biocompatibility Results

**Figure 7:**
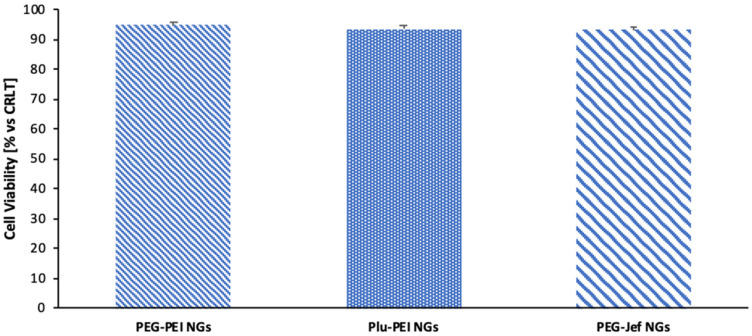
Microglia viability after incubation for 3 days in the presence of nanogels.

The synthetized nanogels were evaluated as biocompatible systems in a biological environment. In vitro cytotoxicity tests were realized by culturing microglia for three days, while sharing the medium with nanogels, and then measured with MTS assay. The quantity of nanogel dissolved in the cell medium is in accordance with the concentration commonly used in pharmacological treatments and in biomedical applications [34]. In Figure 7, cell viability in presence of PEG-PEI NGs, Plu-PEI NGs, and PEG-Jef NGs is reported. A similar trend can be observed in all samples and is comparable to the cells trend without nanogel treatment (CTRL).

## 4. Conclusions

In this research, we have proposed three nanogel core formulations starting from PEG-PEI NGs and studying the role of the polymeric chains that constitute their framework. Their characterization, drug delivery tests, and biocompatibility assays have shown their potentiality as drug carriers. In particular, in each case, it is possible to observe that the change of one polymer does not affect the biocompatibility of the system, but at the same time, the tuning of the formulation can determine specific features on the final device in terms of size, surface potential, as well as drug loading and release ability. This tunable behavior can be pivotal in many cases and applications where, for example, a specific control of the drug release is needed.

Further developments will certainly be focused on the study of new formulations, investigating their potentiality and exploring more tuning possibilities for the composition of NGs frameworks.

## Figures and Tables

**Table 1:** Physical characterization of nanogel formulations through dynamic light scattering analyses.

Nanogel	Diameter (nm)	Polydispersity Index (−)	ζ-Potential (mV)
PEG-PEI NGs	180	0.15	0.01
Plu-PEI NGs	103	0.271	−0.1
PEG-Jef NGs	165	0.252	−0.08

**Table 2:** Loading percentages of hydrophilic drug mimetics (sodium fluorescein (SF) and rhodamine B (RhB)) and hydrophobic drug mimetics (pyrene) in nanogels.

Nanogel	SF Loading (%)	RhB Loading (%)	Pyrene Loading (%)
PE-PEI NGs	51	56	96
Plu-PEI NGs	58	59	92
PEG-Jef NGs	69	76	98

**Table 3:** Diffusion coefficient (D) of the drug mimetics (SF and RhB) that allow to evaluate the influence of the framework on the drug release.

Nanogel	D-SF (m^2^/s)	D-RhB (m^2^/s)
PEG-PEI NGs	2.00 × 10^−9^	2.00 × 10^−9^
Plu-PEI NGs	2.70 × 10^−9^	3.20 × 10^−9^
PEG-Jef NGs	3.30 × 10^−9^	3.00 × 10^−9^

## Supplementary Materials

Supplementary materials can be found at https://www.mdpi.com/1422-0067/21/18/6621/s1. In supplementary document more details about the synthesis of functionalized polymers and nanogels can be found. Moreover, the NMR spectra are reported with higher resolution.

## Abbreviations

CDI	1,1′-carbonyldiimidazole
DLS	Dynamic Light Scattering
Jef	Jeffammina (Elastamine^®^ Re-2000 amine)
NGs	NanoGels
PBS	Phosphate-Buffered Saline solution
PDI	PolyDispersity Index
PEG	PolyEthylene Glycol
PEI	PolyEthylImine
RhB	Rhodamine B
Plu	Pluronic F-127
SF	Sodium Fluorescein
