# Supplementary material for: Influence of the Core Formulation on Features and Drug Delivery Ability of Carbamate-Based Nanogels
Source: Int J Mol Sci. 2020 Sep 10;21(18):6621. doi: 10.3390/ijms21186621 (PMC7555386; doi:10.3390/ijms21186621)
Supplement: Supplementary file 1 [file ijms-21-06621-s001.pdf]

International Journal of *Molecular Sciences*

Supplementary Materials

1. Synthesis of PEG-CDI (1)

The activation of PEG_{8000} with 1,1′-carbonyldiimidazole (CDI) was performed following the procedure illustrated by Vinogradov and coworkers [1]. In details, PEG 8000 (2 g, 0.25 mmol) was dissolved in acetonitrile (20 mL) and then CDI (405 mg, 2.5 mmol) was added to the solution, left under stirring for 17 h at 40 °C. The solvent was evaporated under pressure and the residue was dissolved in distilled water (20mL), purified through dialysis against distilled water (membrane Mw cutoff = 3500 Da) and lyophilized. The modified PEG was confirmed by ^{1}H-NMR analysis. ^{1}H-NMR (400 MHz, $CDCl_3$): δ 8.15 (s, 2H), 7.44 (s, 2H), 7.07 (s, 2H), 4.58 – 4.53 (m, 4H), 3.82 (dt, J = 9.9, 5.0 Hz, 12H), 3.64 (s, 1034H), 3.48 – 3.44 (m, 6H).

2. Synthesis of Propargyl-PEI (2)

Referring to the method proposed by Zhao and coworkers [2], PEI (average Mn 2500 Da, 300 mg, 0.12 mmol) was dissolved in methanol (4 mL) and propargyl bromide solution in 80% toluene (113 µl, 1.2 mmol) was added dropwise at 0 °C. The resulting solution was stirred for 48 h at room temperature. Then, it was concentrated under vacuum and dissolved in distilled water (4 mL) and added NaOH 1 M to adjust pH at 9.5. The mixture was dialyzed (membrane Mw cutoff = 100-500 Da) against distilled water and then lyophilized to obtain the product, which was confirmed by ^{1}H-NMR. ^{1}H-NMR (400MHz, D_2O): δ 3.53 (s, 0.35H), 2.76 (s, 2H).

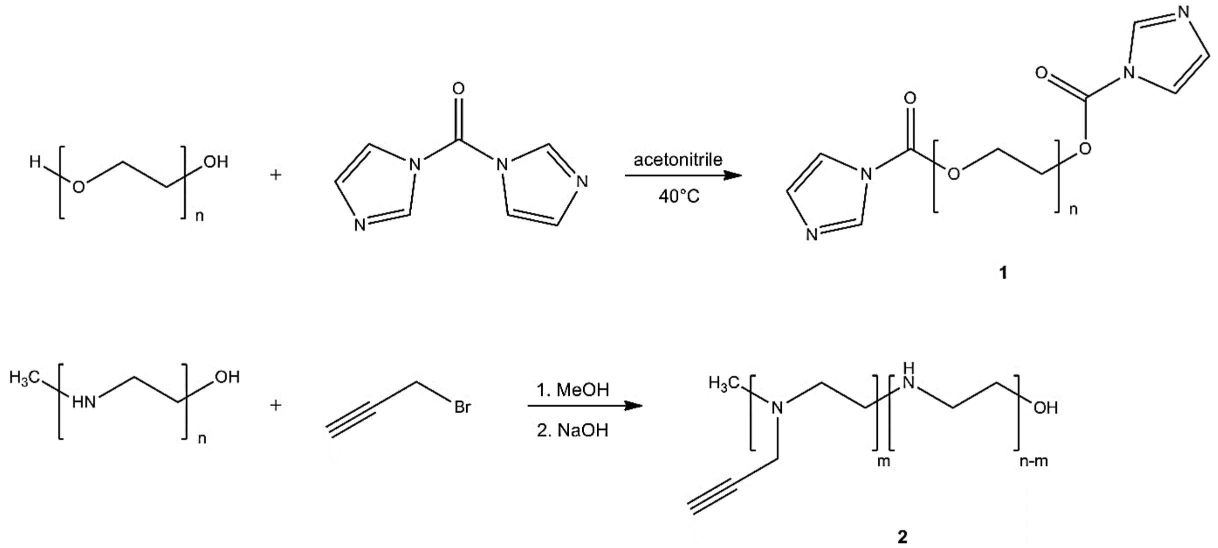

Figure S1. PEG and PEI functionalizations: PEG was reacted with CDI giving product 1; PEI was reacted with propargyl bromide obtaining the alkyne modified polymer 2.

3. Synthesis of Rhodamine-azide (3)

Rhodamine B base was linked to azido group following the method discussed by Wei and coworkers [3]. Synthetized 4-azidobutanol (100 mg, 0.87 mmol) and Rhodamine B (257 mg, 0.58 mmol) were dissolved in CH_2Cl_2 (10 mL) and then 1-ethyl-3-(3-dimethylaminopropyl) carbodiimide hydrochloride (135 mg,0.87 mmol) and 4-(dimethylamino)pyridine (71 mg, 0.58 mmol) were added. The mixture was stirred for 24 h at room temperature and washed, successively, with HCl 1 N (3 × 10 mL), $NaHCO_3$ 1 N (3 × 10 mL) and NaCl ss (30 mL). The resulting organic phase was dried over anhydrous sodium sulfate and concentrated under vacuum. The obtained residue was dissolved in THF (5 mL) and ethyl ether (20 mL) was added to precipitate the final compound 3, which was collected by vacuum filtration and dried under vacuum. The product was characterized by ^{1}H-

NMR. ^{1}H-NMR (400 MHz, $CDCl_3$): δ 7.08 (d, J = 9.5 Hz, 2H), 6.95 (dd, J = 9.5, 2.5 Hz, 2H), 6.84 (d, J = 2.5 Hz, 2H), 6.54 (d, J = 8.9 Hz, 1H), 6.44 (d, J = 2.6 Hz, 1H), 6.32 (dd, J = 8.9, 2.6 Hz, 1H), 4.05 (t, J = 6.2 Hz, 2H), 3.66 (q, J = 7.1 Hz, 4H), 3.35 (q, J = 7.1 Hz, 4H), 3.20 (t, J = 6.6 Hz, 2H), 1.60–1.41 (m, 4H), 1.34 (t, J=7.1 Hz, 6H), 1.17 (t, J = 7.1 Hz, 6H).

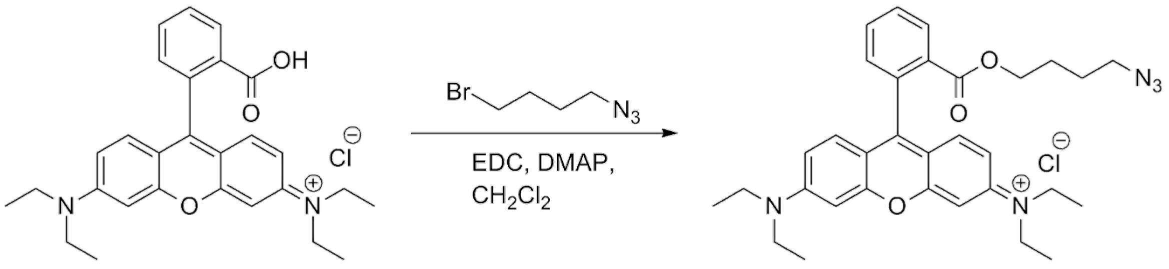

3

Figure S2. Formation of the rhodamine azide **3.**

4. Click Reaction of PEI-Rhodamine or PEI-Cy5 (4)

Modified PEI **2** (200 mg, 0.08 mmol) was dissolved in distilled water (10 mL) and then **3** (43 mg, 0.08 mmol) or Cy5-azide (used as purchased, 8 mg, 0.008 mmol) was added. To this solution, copper iodide (3 mg, 0.016 mmol) and sodium ascorbate (3.2 mg, 0.016 mmol) were added and the resulting reaction system was allowed to stir for 24 h at 50 °C. Subsequently, it was cooled at 25 °C and dialyzed (membrane: "Biotech CE tubing" with Mw cutoff = 100-500 Da) against aqueous solution at pH 5, consisting of distilled water (2000 mL), sodium chloride (11.2 g) and HCl 37% *w/w* (4 drops). Dialysis occurred for two days, with daily replacement of the dialysis solution. Finally, the mixture was lyophilized, giving a purple solid, in the case of RhB, or a light blue solid with Cy5.

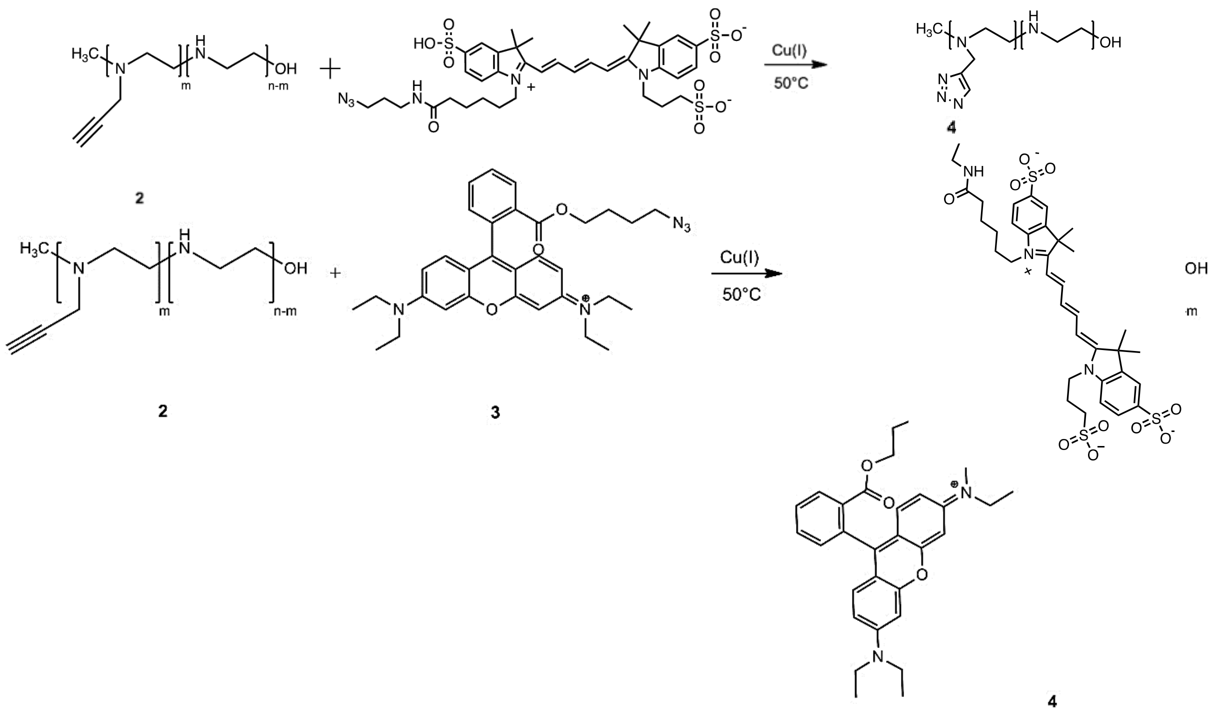

Figure S3. Click reaction between PEI **2** and the dye (with Cy5 above, Rhodamine B below) to give product **4**.

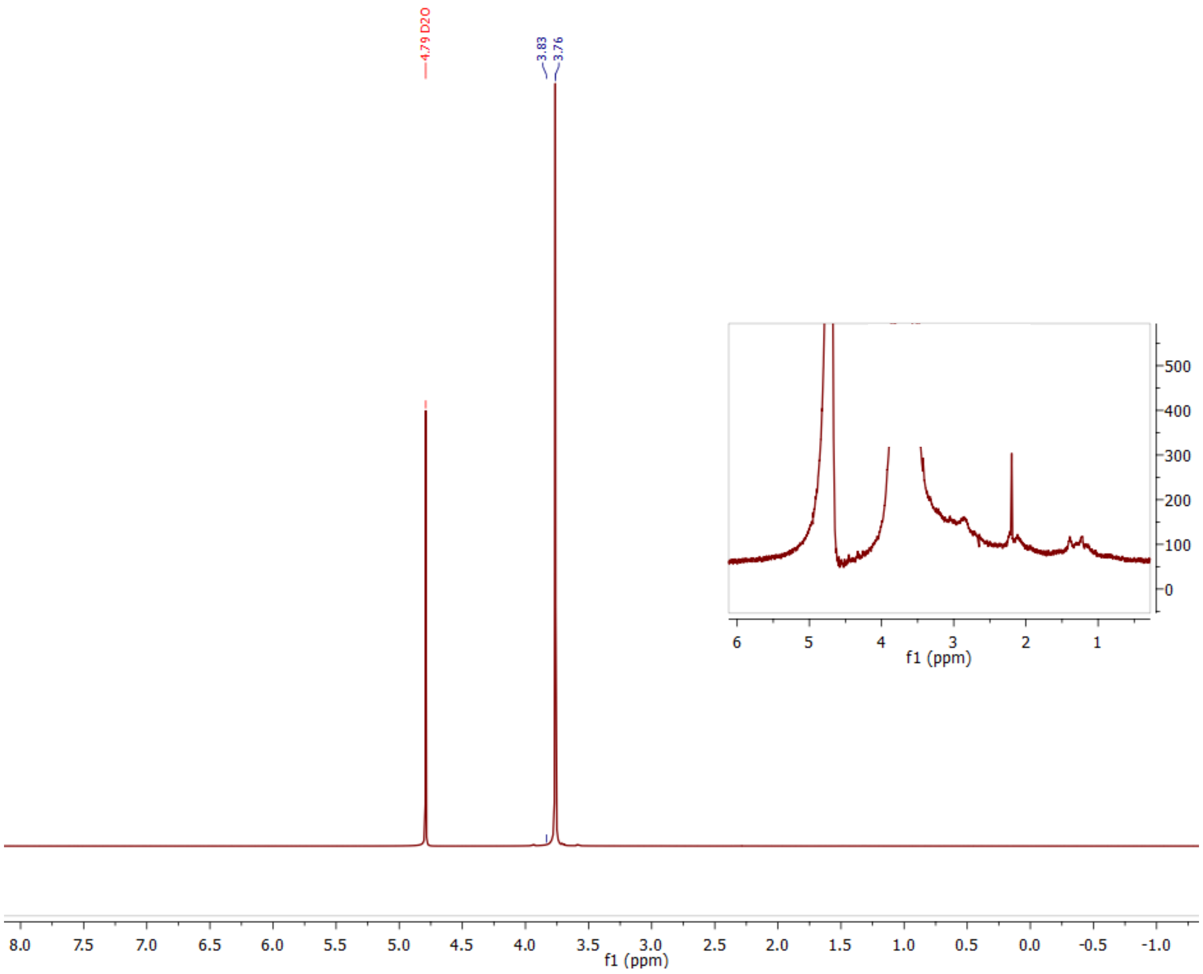

Figure S4. ^1H-NMR spectrum of PEG-PEI NGs.

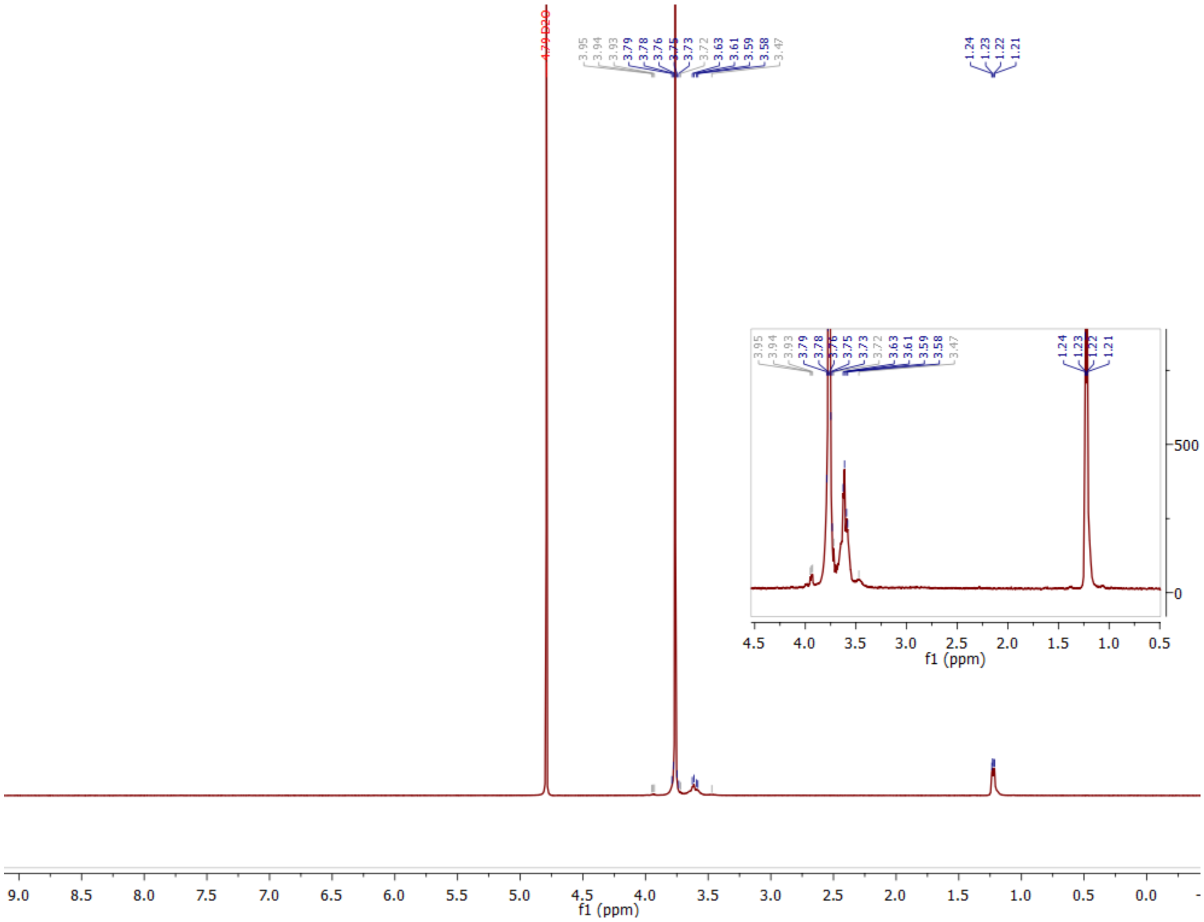

Figure S5. ^{1}H-NMR spectrum of Plu-PEI NGs.

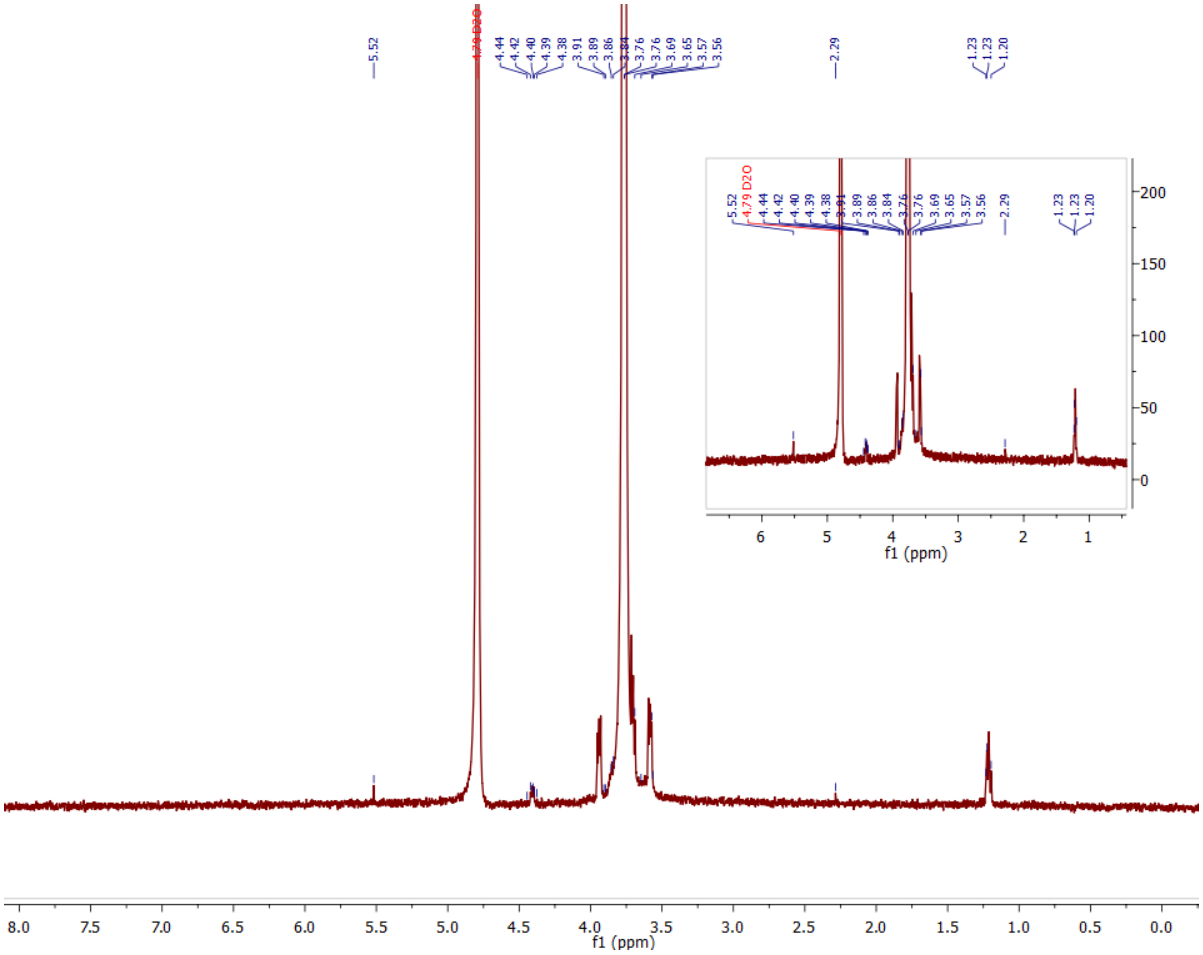

Figure S6. ^{1}H-NMR spectrum of PEG-Jef NGs.

References

1. Vinogradov, S.; Batrakova, E.; Kabanov, A. Poly(ethylene glycol)-polyethyleneimine NanoGel(TM) particles: Novel drug delivery systems for antisense oligonucleotides. *Colloids Surf. B* **1999**, *16*, 291–304, 1999.
2. Zhao, N.; Roesler, S.; Kissel, T. Synthesis of a new potential biodegradable disulfide containing poly(ethylene imine)-poly(ethylene glycol) copolymer cross-linked with click cluster for gene delivery/ *Int. J. Pharm.* **2011**, *411*, 197–205.
3. Wei, X.; Chen, W.; Chen, X.; Russell, T.P. Disorder-to-order transition of diblock copolymers induced by alkyne/azide click chemistry *Macromolecules* **2010**, *43*, 6234–6236.
